# Phylogenomic analyses data of the avian phylogenomics project

**DOI:** 10.1186/s13742-014-0038-1

**Published:** 2015-02-12

**Authors:** Erich D Jarvis, Siavash Mirarab, Andre J Aberer, Bo Li, Peter Houde, Cai Li, Simon Y W Ho, Brant C Faircloth, Benoit Nabholz, Jason T Howard, Alexander Suh, Claudia C Weber, Rute R da Fonseca, Alonzo Alfaro-Núñez, Nitish Narula, Liang Liu, Dave Burt, Hans Ellegren, Scott V Edwards, Alexandros Stamatakis, David P Mindell, Joel Cracraft, Edward L Braun, Tandy Warnow, Wang Jun, M Thomas Pius Gilbert, Guojie Zhang, The Avian Phylogenomics Consortium

**Affiliations:** Department of Neurobiology, Howard Hughes Medical Institute and Duke University Medical Center, Durham, NC 27710 USA; Department of Computer Science, The University of Texas at Austin, Austin, TX 78712 USA; Scientific Computing Group, Heidelberg Institute for Theoretical Studies, Heidelberg, Germany; China National GeneBank, BGI-Shenzhen, Shenzhen, 518083 China; College of Medicine and Forensics, Xi’an Jiaotong University, Xi’an, 710061 China; Centre for GeoGenetics, Natural History Museum of Denmark, University of Copenhagen, Øster Voldgade 5-7, 1350 Copenhagen, Denmark; Department of Biology, New Mexico State University, Las Cruces, NM 88003 USA; School of Biological Sciences, University of Sydney, Sydney, NSW 2006 Australia; Department of Ecology and Evolutionary Biology, University of California Los Angeles, Los Angeles, CA 90095 USA; Department of Biological Sciences, Louisiana State University, Baton Rouge, LA 70803 USA; CNRS UMR 5554, Institut des Sciences de l’Evolution de Montpellier, Université Montpellier II, Montpellier, France; Department of Evolutionary Biology, Uppsala University, SE-752 36 Uppsala, Sweden; Biodiversity and Biocomplexity Unit, Okinawa Institute of Science and Technology Onna-son, Okinawa, 904-0495 Japan; Department of Statistics and Institute of Bioinformatics, University of Georgia, Athens, 30602 USA; Department of Genomics and Genetics, The Roslin Institute and Royal (Dick) School of Veterinary Studies, University of Edinburgh, Easter Bush Campus, Midlothian, EH25 9RG UK; Department of Organismic and Evolutionary Biology and Museum of Comparative Zoology, Harvard University, Cambridge, MA USA; Institute of Theoretical Informatics, Department of Informatics, Karlsruhe Institute of Technology, D- 76131 Karlsruhe, Germany; Department of Biochemistry & Biophysics, University of California, San Francisco, CA 94158 USA; Department of Ornithology, American Museum of Natural History, New York, NY 10024 USA; Department of Biology and Genetics Institute, University of Florida, Gainesville, FL 32611 USA; Department of Biology, University of Copenhagen, Ole Maaløes Vej 5, 2200 Copenhagen, Denmark; Princess Al Jawhara Center of Excellence in the Research of Hereditary Disorders, King Abdulaziz University, Jeddah, 21589 Saudi Arabia; Macau University of Science and Technology, Avenida Wai long, Taipa, Macau, 999078 China; Department of Medicine, University of Hong Kong, Hong Kong, Hong Kong; Trace and Environmental DNA Laboratory Department of Environment and Agriculture, Curtin University, Perth, WA 6102 Australia; Centre for Social Evolution, Department of Biology, Universitetsparken 15, University of Copenhagen, DK-2100 Copenhagen, Denmark

**Keywords:** Avian genomes, Phylogenomics, Sequence alignments, Species tree, Gene trees, Indels, Transposable elements

## Abstract

**Background:**

Determining the evolutionary relationships among the major lineages of extant birds has been one of the biggest challenges in systematic biology. To address this challenge, we assembled or collected the genomes of 48 avian species spanning most orders of birds, including all Neognathae and two of the five Palaeognathae orders. We used these genomes to construct a genome-scale avian phylogenetic tree and perform comparative genomic analyses.

**Findings:**

Here we present the datasets associated with the phylogenomic analyses, which include sequence alignment files consisting of nucleotides, amino acids, indels, and transposable elements, as well as tree files containing gene trees and species trees. Inferring an accurate phylogeny required generating: 1) A well annotated data set across species based on genome synteny; 2) Alignments with unaligned or incorrectly overaligned sequences filtered out; and 3) Diverse data sets, including genes and their inferred trees, indels, and transposable elements. Our total evidence nucleotide tree (TENT) data set (consisting of exons, introns, and UCEs) gave what we consider our most reliable species tree when using the concatenation-based ExaML algorithm or when using statistical binning with the coalescence-based MP-EST algorithm (which we refer to as MP-EST*). Other data sets, such as the coding sequence of some exons, revealed other properties of genome evolution, namely convergence.

**Conclusions:**

The Avian Phylogenomics Project is the largest vertebrate phylogenomics project to date that we are aware of. The sequence, alignment, and tree data are expected to accelerate analyses in phylogenomics and other related areas.

**Electronic supplementary material:**

The online version of this article (doi:10.1186/s13742-014-0038-1) contains supplementary material, which is available to authorized users.

## Data description

Here we present FASTA files of loci, sequence alignments, indels, transposable elements, and Newick files of gene trees and species trees used in the Avian Phylogenomics Project [1-4]. We also include scripts used to process the data. The 48 species from which we collected these data span the phylogeny of modern birds, including representatives of all Neognathae (Neoaves and Galloanseres) and two of the five Palaeognathae orders (Table 1) [5-7].

**Table 1:** **Genomes used in the avian phylogenomics project**

**Species**	**English name**	**BioProject ID**	**GigaScience**
*Acanthisitta chloris*	Rifleman	PRJNA212877	http://dx.doi.org/10.5524/101015
*Anas platyrhynchos*	Pekin Duck	PRJNA46621	http://dx.doi.org/10.5524/101001
*Antrostomus carolinensis*	Chuck-will’s-widow	PRJNA212888	http://dx.doi.org/10.5524/101019
*Apaloderma vittatum*	Bar-tailed Trogon	PRJNA212878	http://dx.doi.org/10.5524/101016
*Aptenodytes forsteri*	Emperor Penguin	PRJNA235982	http://dx.doi.org/10.5524/100005
*Balearica regulorum*	Grey Crowned-crane	PRJNA212879	http://dx.doi.org/10.5524/101017
*Buceros rhinoceros*	Rhinoceros Hornbill	PRJNA212887	http://dx.doi.org/10.5524/101018
*Calypte anna*	Anna’s Hummingbird	PRJNA212866	http://dx.doi.org/10.5524/101004
*Cariama cristata*	Red-legged Seriema	PRJNA212889	http://dx.doi.org/10.5524/101020
*Cathartes aura*	Turkey Vulture	PRJNA212890	http://dx.doi.org/10.5524/101021
*Chaetura pelagica*	Chimney Swift	PRJNA210808	http://dx.doi.org/10.5524/101005
*Charadrius vociferus*	Killdeer	PRJNA212867	http://dx.doi.org/10.5524/101007
*Chlamydotis macqueenii*	MacQueen’s Bustard	PRJNA212891	http://dx.doi.org/10.5524/101022
*Colius striatus*	Speckled Mousebird	PRJNA212892	http://dx.doi.org/10.5524/101023
*Columba livia*	Pigeon	PRJNA167554	http://dx.doi.org/10.5524/100007
*Corvus brachyrhynchos*	American Crow	PRJNA212869	http://dx.doi.org/10.5524/101008
*Cuculus canorus*	Common Cuckoo	PRJNA212870	http://dx.doi.org/10.5524/101009
*Egretta garzetta*	Little Egret	PRJNA232959	http://dx.doi.org/10.5524/101002
*Eurypyga helias*	Sunbittern	PRJNA212893	http://dx.doi.org/10.5524/101024
*Falco peregrinus*	Peregrine Falcon	PRJNA159791	http://dx.doi.org/10.5524/101006
*Fulmarus glacialis*	Northern Fulmar	PRJNA212894	http://dx.doi.org/10.5524/101025
*Gallus gallus*	Chicken	PRJNA13342	N.A.
*Gavia stellata*	Red-throated Loon	PRJNA212895	http://dx.doi.org/10.5524/101026
*Geospiza fortis*	Medium Ground-finch	PRJNA156703	http://dx.doi.org/10.5524/100040
*Haliaeetus albicilla*	White-tailed Eagle	PRJNA212896	http://dx.doi.org/10.5524/101027
*Haliaeetus leucocephalus*	Bald Eagle	PRJNA237821	http://dx.doi.org/10.5524/101040
*Leptosomus discolor*	Cuckoo-roller	PRJNA212897	http://dx.doi.org/10.5524/101028
*Manacus vitellinus*	Golden-collared Manakin	PRJNA212872	http://dx.doi.org/10.5524/101010
*Meleagris gallopavo*	Turkey	PRJNA42129	N.A.
*Melopsittacus undulatus*	Budgerigar	PRJNA72527	http://dx.doi.org/10.5524/100059
*Merops nubicus*	Carmine Bee-eater	PRJNA212898	http://dx.doi.org/10.5524/101029
*Mesitornis unicolor*	Brown Mesite	PRJNA212899	http://dx.doi.org/10.5524/101030
*Nestor notabilis*	Kea	PRJNA212900	http://dx.doi.org/10.5524/101031
*Nipponia nippon*	Crested ibis	PRJNA232572	http://dx.doi.org/10.5524/101003
*Opisthocomus hoazin*	Hoatzin	PRJNA212873	http://dx.doi.org/10.5524/101011
*Pelecanus crispus*	Dalmatian Pelican	PRJNA212901	http://dx.doi.org/10.5524/101032
*Phaethon lepturus*	White-tailed Tropicbird	PRJNA212902	http://dx.doi.org/10.5524/101033
*Phalacrocorax carbo*	Great Cormorant	PRJNA212903	http://dx.doi.org/10.5524/101034
*Phoenicopterus ruber*	American Flamingo	PRJNA212904	http://dx.doi.org/10.5524/101035
*Picoides pubescens*	Downy Woodpecker	PRJNA212874	http://dx.doi.org/10.5524/101012
*Podiceps cristatus*	Great Crested Grebe	PRJNA212905	http://dx.doi.org/10.5524/101036
*Pterocles gutturalis*	Yellow-throated Sandgrouse	PRJNA212906	http://dx.doi.org/10.5524/101037
*Pygoscelis adeliae*	Adelie Penguin	PRJNA235983	http://dx.doi.org/10.5524/100006
*Struthio camelus*	Common Ostrich	PRJNA212875	http://dx.doi.org/10.5524/101013
*Taeniopygia guttata*	Zebra Finch	PRJNA17289	N.A.
*Tauraco erythrolophus*	Red-crested Turaco	PRJNA212908	http://dx.doi.org/10.5524/101038
*Tinamus guttatus*	White-throated Tinamou	PRJNA212876	http://dx.doi.org/10.5524/101014
*Tyto alba*	Barn Owl	PRJNA212909	http://dx.doi.org/10.5524/101039

Listed are the scientific species name, English name, BioProject ID in the NCBI database for each genome (http://www.ncbi.nlm.nih.gov/bioproject), and *GigaScience* deposited genome sequences and raw reads. Full details are in [1,2].

### Explanation of various data sets used to infer gene and species trees

Here we describe each locus data set in brief. Additional details are provided in Jarvis et al. [1].

#### 8295 protein-coding exon gene set

This is an exon-coding sequence data set of 8295 genes based on synteny-defined orthologs we identified and selected from the assembled genomes of chicken and zebra finch [8,9]. We required these loci to be present in at least 42 of the 48 avian species and outgroups, which allowed for missing data due to incomplete assemblies. To be included in the dataset, the exons in each genome assembly had to be 30% or more of the full-length sequence of the chicken or zebra finch ortholog. Annotated untranslated regions (UTRs) were trimmed off to remove non-coding sequence, in order to infer a coding-only sequence phylogeny. We note that 44 genes were identified with various problems such as gene annotation issues, and we removed them in the phylogenetic analyses. However, we provide them here in the unfiltered alignments.

#### 8295 protein amino acid alignment set

These are alignments of the translated peptide sequences for the 8295 protein-coding gene data set.

#### 2516 intron gene set

This is an orthologous subset of introns from the 8295 protein-coding genes among 52 species (includes outgroups). Introns with conserved annotated exon-intron boundaries between chicken and another species (±1 codon) were chosen. We filtered out introns with length < 50 bp or intron length ratio > 1.5 between chicken and another species or another species and chicken. This filtering resulted in a conservative subset of introns that could be reliably identified and aligned.

#### 3679 UCE locus set

This is the ultraconserved element (UCE) data set with 1000 bp flanking sequence at the 3′ and 5′ ends. The UCE dataset was filtered to remove overlap with the above exon and intron data sets, other exons and introns in the chicken genome assembly version 3, and overlapping sequences among the UCEs. The source UCE sequences used to search the genomes were determined from sequence capture probes [10-12] aligned to each avian genome assembly. Unlike the exon and intron data sets, we required that all 42 avian species and the alligator outgroup contain the UCEs. We found this requirement to be sufficient, because the central portions of UCEs are highly conserved across all species.

#### High and low variance introns and exons

These four data sets represent the 10% subsets of the 8295 exons and their associated introns when available (i.e. from the same genes) that had the highest and lowest variance in GC3 (third codon position) content across species. To calculate GC3 variance, we first calculated GC3 for each ortholog in each species, and then we used the correlation coefficient R to calculate variance in GC3 for each species. Orthologs were ranked by their GC3 variance and we selected the top and bottom 10% for analyses.

#### Supergenes

These are the concatenated sets of loci from various partitions of the TENT dataset (exons, introns, and UCEs described above), brought together using the statistical binning approach. The statistical binning approach put together sets of loci that were deemed “combinable”. Two genes were considered combinable if their respective gene trees had no pairs of incompatible branches that had bootstrap support above a 50% threshold. Alignments of genes in the same bin were concatenated to form supergenes, but boundaries of genes were kept so that a gene-partitioned phylogenetic analysis could be performed on each supergene.

#### Whole genome alignment

Whole genome alignments were first created by a LASTZ + MULTIZ alignment [13,14] (http://www.bx.psu.edu/miller_lab/) across all 48 bird species and outgroups using individual chromosomes of the chicken genome as the reference (initial alignment 392,719,329 Mb). They were filtered for segments with fewer than 42 avian species (>5 missing bird species) and aberrant sequence alignments. The individual remaining segments of the MULTIZ alignment were realigned with MAFFT. We did not use SATé + MAFFT due to computational challenges (too much input/output was required).

#### Indel dataset

5.7 million insertions and deletions (indels) were scored as binary characters locus by locus from the same intron, exon, and UCE alignments as used in the TENT data set on the principle of simple indel coding using 2Xread [15,16] and then concatenated. Coding was verified using GapCoder [17] and by visual inspection of alignments for a small subset of data. Intron indels were scored on alignments that excluded non-avian outgroups (48 taxa), UCE indels were scored on alignments that included Alligator (49 taxa), and exons were scored on alignments that included all non-avian outgroups (52 taxa). Individual introns of the same gene were scored independently to avoid creating artifactual indels between concatenated intron or whole genome segments, whereas exons were concatenated as complete unigenes before scoring. For exons, indels >30 bp were excluded to avoid scoring missing exons as indels.

#### Transposable element markers

These are 61 manually curated presence/absence loci of transposable elements (TEs) present in the Barn Owl genome that exhibit presence at orthologous positions in one or more of the other avian species. The TE markers were identified by eye after a computational screening of 3,671 TguLTR5d retroposon insertions from the Barn Owl. For each TguLTR5d locus, we conducted BLASTn searches of TE-flanking sequences (1 kb per flank) against the remaining avian species and generated multispecies sequence alignments using MAFFT [18]. Redundant or potentially paralogous loci were excluded from analysis and the remaining marker candidates were carefully inspected using strict standard criteria for assigning presence/absence character states [19-21].

### FASTA files of loci datasets in alignments

We provide the above loci data sets as FASTA files of both unfiltered and filtered sequence alignments. The alignments were filtered for aberrant over- and under-aligned sequences, and for the presence of the loci in 42 of the 48 avian species. All multiple sequence alignments were performed in two rounds. The first round was used to find contiguous portions of sequences that we identified as aberrant, and the second round was used to realign the filtered sequences. We used SATé [22,23] combined with either MAFFT [18] or PRANK [24] alignment algorithms, depending on the limitations of working with large datasets. Alignments without and with outgroups are made available.

### Filtered loci sequence alignments

#### Exon loci alignments

These are filtered alignments of exons from 8295 genes. Of these 8295, there were 42 genes that were identified to have annotation issues and we removed them from the phylogenetic analyses (the list is provided in the file FASTA_files_of_loci_datasets/Filtered_sequence_alignments/8295_Exons/42-exon-genes-removed.txt). Two more genes were removed because a gene tree could not be estimated for them. The first round of alignment was performed using SATé + PRANK, and the second round was performed using SATé + MAFFT. Before alignment, the nucleotide sequences were converted to amino acid sequences, and then reverted back to nucleotide sequences afterwards.

8295 Exons

- 42-exon-genes-removed.txt: list of 42 genes removed due to various issues
- pep2cds-filtered-sate-alignments-noout.tar.gz: DNA alignments (Amino acid alignments translated to DNA) without outgroups
- pep2cds-filtered-sate-alignments-original.zip: DNA alignments (Amino acid alignments translated to DNA) with outgroups included

8295 Amino Acids

- pep-filtered-sate-alignments-noout.tar.gz: Amino acid alignments with outgroups removed
- pep-filtered-sate-alignments-original.zip: Amino acid alignments with outgroups included

#### Intron loci alignments

These are filtered alignments of introns from 2516 genes. Both rounds of alignment were performed using SATé + MAFFT, because SATé + PRANK was too computationally expensive on long introns.

2516 Introns

- introns-filtered-sate-alignments-with-and-without-outgroups.tar.gz: Includes both alignments with and without outgroups

#### UCE loci alignments

These are alignments of UCEs and their surrounding 1000 bp from 3769 loci after filtering. Both rounds of alignment were performed using SATé + MAFFT.

3769 UCE + 1000 flanking bp

- uce-probes-used.fasta.gz: Probes targeting UCE loci shared among vertebrate taxa.
- uce-raw-genome-slices-of-probe-matches.tar: Probe + flank slices around locations matching probes targeting UCE loci.
- uce-raw-lastz-results-of-probe-matches.tar: LASTZ results of mapping probes onto genome assemblies.
- uce-assembled-loci-from-probe-matches.tar: UCE loci assembled from probe + flank slices from each genome.
- uce-filtered-alignments-w-gator.tar.gz: UCE individual alignments without outgroups
- uce-filtered-alignments-without-gator.tar.gz: UCE individual alignments with outgroups

#### Supergenes generated from statistical binning

These are concatenated alignments for each of our 2022 supergene alignments. We note that although supergenes are concatenated loci, we estimated supergene trees using partitioned analyses where each gene was put in a different partition. Thus, we also provide the boundaries between genes in text files (these can be directly used as partition input files to RAxML).

- supergene-alignments.tar.bz2: supergene alignments with partition files showing genes put in each bin and their boundaries in the concatenated alignment

### Unfiltered loci sequence alignments

These are individual loci alignments of the above data sets, before filtering.

Amino.Acid.unfiltered

- pep-unfiltered-alignments-original.zip: unfiltered SATé + Prank alignments used for the filtering step

Exon.c123.unfiltered:

- pep2cds-unfiltered-alignemtns-original.zip: unfiltered SATé + Prank alignments used for the filtering step

Intron.unfiltered

- introns-unfiltered-alignments-original.zip: intron SATé alignments before filtering with outgroups included
- introns-unfiltered-alignments-noout.zip: intron SATé alignments before filtering with outgroups included

UCE.unfiltered

- uce-unfiltered-alignments-w-gator.tar.gz: UCE alignments before filtering with alligator outgroup

WGT.unfiltered

- These are uploaded as part of the comparative genomics paper [2] data note [25], and a link is provided here https://github.com/gigascience/paper-zhang2014.

### FASTA files of concatenated datasets in alignments

We provide FASTA files of concatenated sequence alignments of the above filtered loci datasets. These are concatenated alignments that were used in the ExaML and RAxML analyses [3].

#### Concatenated alignments used in ExaML analyses

- Exon.AminoAcid.ExaML.partitioned
- Exon.c123. ExaML.partitioned
- Exon.c123. ExaML.unpartitioned
- Exon.c1.ExaML.unpartitioned
- Exon.c2.ExaML.unpartitioned
- Exon.c12.ExaML.unpartitioned
- Exon.c123-RY.ExaML.unpartitioned
- Exon.c3.ExaML.unpartitioned
- Intron
- TEIT.RAxML
- TENT + c3.ExaML
- TENT + outgroup.ExaML
- TENT.ExaML.100%
- TENT.ExaML.25%
- TENT.ExaML.50%
- TENT.ExaML.75%
- WGT.ExaML

#### Concatenated alignments used in RAxML analyses

UCE concatenated alignments with and without the alligator

- uce-filtered-alignments-w-gator-concatenated.phylip.gz
- uce-filtered-alignments-without-gator-concatenated.phylip.gz

#### Clocklike exon alignment

Concatenated c12 (1st + 2nd codons) DNA sequence alignments from the 1156 clocklike genes were used for the dating analyses. These are alignments of the first and second codon positions of clock-like genes among the 8295 exon orthologs:

- c12.DNA.alignment.1156.clocklike.zip
- c12.DNA.alignment.1156.clocklike.txt
- c12.DNA.alignment.clocklike.readme.txt
- c12.DNA.alignment.clocklike.txt.zip

#### High and low variance exons and their associated introns

- High variance exons:

- Exon.heterogeneous.c123
- Exon.heterogenous.c12

- Low variance exons:

- Exon.homogeneous.c123.
- Exon.homogenous.c12

- High variance introns: These are heterogenous introns

- concatIntronNooutMSAlow.fasta.gz

- Low variance introns: These are homogenous introns

- concatIntronNooutMSAhigh.fasta.gz

#### Indel sequence alignments

This is a concatenated alignment of indels from exons, introns, and UCEs. A README file describes the content.

### Transposable element markers

- owl_TE_marker_Table.txt

### Species and gene tree files

Species trees (Newick format) were generated with either RAxML, an improved ExaML version for handling large alignments, or MP-EST* [4]. We deposit both the maximum likelihood and bootstrap replicate trees.

#### Newick files for 32 species trees using different genomic partitions and methods

- Exon.AminoAcid.ExaML.partitioned.tre
- Exon.c123.ExaML.partitioned.tre
- Exon.c123.ExaML.unpartititoned.tre
- Exon.c123-RY.ExaML.unpartitioned.tre
- Exon.c12.ExaML.partitioned.tre
- Exon.c12.ExaML.unpartitioned.tre
- Exon.c1.ExaML.unpartitioned.tre
- Exon.c2.ExaML.unpartitioned.tre
- Exon.c3.ExaML.unpartitioned.tre
- Exon.RAxML.heterogenous.c123.tre
- Exon.RAxML.heterogenous.c12.tre
- Exon.RAxML.homogenous.c123.tre
- Exon.RAxML.homogenous.c12.tre
- Intron.RAxML.heterogenous.tre.txt
- Intron.RAxML.homogenous.tre.txt
- Intron.RAxML.partitioned.tre
- Intron.RAxML.unpartitioned.tre
- Intron.MP-EST.binned.tre
- Intron.MP-EST.unbinned.tre
- TEIT.RAxML.tre
- TENT + c3.ExaML.tre
- TENT + outgroup.ExaML.tre
- TENT.ExaML.100%.tre
- TENT.ExaML.25%.tre
- TENT.ExaML.50%.tre
- TENT.ExaML.75%.tre
- UCE.RAxML.unpartitioned.tre
- WGT.ExaML.alternative.tre
- WGT.ExaML.best.tree

#### Newick files of the 11 timetrees (chronograms)

- Chronogram01.TENT.ExAML.tre
- Chronogram02.TENT.ExAML.max865.tre
- Chronogram03.TENT.ExAML.Allig247.tre
- Chronogram04.TENT.ExAML.no-outgroup.tre
- Chronogram05.TENT.ExAML.no-outgroup.max865.tre
- Chronogram06.TENT.MP-EST.tre
- Chronogram07.WGT.ExAML.alternative.tre
- Chronogram08.WGT.ExAML.best.tre
- Chronogram09.Intron.ExAML.unpartitioned.tre
- Chronogram10.UCE.RAxML.tre
- Chronogram11.Exon.c123.RaXML.partitioned.tre

#### Newick file downloads of gene trees (species abbreviated with 5-letter names)

- ML (bestML) gene trees
- Bootstrap replicates of ML gene trees
- ML (bestML) supergene trees used in MP-EST analyses
- Bootstrap replicates of supergene trees used in MP-EST analyses
- Partition files showing which loci make up which bins for MP-EST analyses

### List of scripts used in avian phylogenomics project

We also deposit the key scripts used in this project in GigaDB, which include:

- Script for filtering amino acid alignments
- Script for filtering nucleotide sequence alignments
- Script for mapping names from 5-letter codes to full names
- Scripts related to indel analyses

We provide readme files in the script directories describing the usage of the scripts.

## Availability and requirements

Project name: Avian Phylogenomic Project scripts

Project home page: https://github.com/gigascience/paper-jarvis2014; also see companion paper home page for related data https://github.com/gigascience/paper-zhang2014

Operating system: Unix

Programming language: R, Perl, python

License: GNU GPL v3.

Any restrictions to use by non-academics: none

## Availability of supporting data

Other data files presented in this data note for the majority of genomes are available in the *GigaScience* repository, GigaDB [26] (Table 1), as well as NCBI (Table 1), ENSEMBL, UCSC, and CoGe databases. ENSEMBL: http://avianbase.narf.ac.uk/index.html UCSC: (http://genome.ucsc.edu/cgi-bin/hgGateway; under vertebrate genomes) CoGe: (https://genomevolution.org/wiki/index.php/Bird_CoGe).

## Additional file

Additional file 1:
**Full author list.**

## Abbreviations

TE: Transposable element
TENT: Total evidence Nucleotide tree
TEIT: Total evidence indel tree
WGT: Whole genome tree
UCE: Ultra conserved element
c123: 1st, 2nd, and 3rd codons of exons
