# Supplementary material for: Phylogenomic analyses data of the avian phylogenomics project
Source: Gigascience. 2015 Feb 12;4:4. doi: 10.1186/s13742-014-0038-1 (PMC4349222; doi:10.1186/s13742-014-0038-1)
Supplement: Additional file 1: — Full author list. [file 13742_2014_38_MOESM1_ESM.doc]

**Full author list (same as (1))**

**Authors**:

Erich D. Jarvis^1*#^, Siavash Mirarab^2*^, Andre J. Aberer^3^, Bo Li^4,5,6^, Peter Houde^7^, Cai Li^4,6^, Simon Y. W. Ho^8^, Brant C. Faircloth^9,10^, Benoit Nabholz^11^, Jason T. Howard^1^, Alexander Suh^12^, Claudia C. Weber^12^, Rute R. da Fonseca^6^, Jianwen Li^4^, Fang Zhang^4^, Hui Li^4^, Long Zhou^4^, Nitish Narula^7,13^, Liang Liu^14^, Ganesh Ganapathy^1^, Bastien Boussau^15^, Md. Shamsuzzoha Bayzid^2^, Volodymyr Zavidovych^1^, Sankar Subramanian^16^, Toni Gabaldón^17,18,19^, Salvador Capella-Gutiérrez^17,18^, Jaime Huerta-Cepas^17,18^, Bhanu Rekepalli^20^, Kasper Munch^21^, Mikkel Schierup^21^, Bent Lindow^6^, Wesley C. Warren^22^, David Ray^23,24,25^, Richard E. Green^26^, Michael Bruford^27^, Xiangjiang Zhan^27,28^, Andrew Dixon^29^, Shengbin Li^30^, Ning Li^31^, Yinhua Huang^31^, Elizabeth P. Derryberry^32,33^, Mads Frost Bertelsen^34^, Frederick H. Sheldon^33^, Robb T. Brumfield^33^, Claudio V. Mello^35,36^, Peter V. Lovell^35^, Morgan Wirthlin^35^, Maria Paula Cruz Schneider^36,37^, Francisco Prosdocimi^36,38^, José Alfredo Samaniego^6^, Amhed Missael Vargas Velazquez^6^, Alonzo Alfaro-Núñez^6^, Paula F. Campos^6^, Bent Petersen^39^, Thomas Sicheritz-Ponten^39^, An Pas^40^, Tom Bailey^41^, Paul Scofield^42^, Michael Bunce^43^, David M. Lambert^16^, Qi Zhou^44^, Polina Perelman^45,46^, Amy C. Driskell^47^, Beth Shapiro^26^, Zijun Xiong^4^, Yongli Zeng^4^, Shiping Liu^4^, Zhenyu Li^4^, Binghang Liu^4^, Kui Wu^4^, Jin Xiao^4^, Xiong Yinqi^4^, Qiuemei Zheng^4^, Yong Zhang^4^, Huanming Yang^48^, Jian Wang^48^, Linnea Smeds^12^, Frank E. Rheindt^49^, Michael Braun^50^, Jon Fjeldsa^51^, Ludovic Orlando^6^, Keith Barker^52^, Knud Andreas Jønsson^51,53,54^, Warren Johnson^55^, Klaus-Peter Koepfli^56^, Stephen O'Brien^57,58^, David Haussler^59^, Oliver A. Ryder^60^, Carsten Rahbek^51,54^, Eske Willerslev^6^, Gary R. Graves^51,61^,, Travis C. Glenn^62^, John McCormack^63^, Dave Burt^64^, Hans Ellegren^12^, Per Alström^65,66^, Scott V. Edwards^67^, Alexandros Stamatakis^3,68^, David P. Mindell^69^, Joel Cracraft^70^, Edward L. Braun^71^, Tandy Warnow^2#^, Wang Jun^48,72,73,74,75#^, M. Thomas P. Gilbert^6,43#^, and Guojie Zhang^4,76#^.

**Affiliations:**

1. Department of Neurobiology, Howard Hughes Medical Institute and Duke University Medical Center, Durham NC 27710 USA
2. Department of Computer Science, The University of Texas at Austin, Austin, TX 78712 USA
3. Scientific Computing Group, Heidelberg Institute for Theoretical Studies, Heidelberg, Germany
4. China National GeneBank, BGI-Shenzhen, Shenzhen, 518083, China
5. College of Medicine and Forensics, Xi'an Jiaotong University Xi'an, 710061, China
6. Centre for GeoGenetics, Natural History Museum of Denmark, University of Copenhagen, Øster Voldgade 5-7, 1350 Copenhagen, Denmark
7. Department of Biology, New Mexico State University, Las Cruces NM 88003 USA
8. School of Biological Sciences, University of Sydney, Sydney NSW 2006, Australia
9. Department of Ecology and Evolutionary Biology, University of California Los Angeles, CA 90095 USA
10. Department of Biological Sciences, Louisiana State University, Baton Rouge, LA, 70803, USA
11. CNRS UMR 5554, Institut des Sciences de l’Evolution de Montpellier, Université Montpellier II Montpellier, France
12. Department of Evolutionary Biology (EBC), Uppsala University, SE-752 36, Uppsala Sweden
13. Biodiversity and Biocomplexity Unit, Okinawa Institute of Science and Technology Onna-son, Okinawa, 904-0495, Japan
14. Department of Statistics and Institute of Bioinformatics, University of Georgia, Athens, 30602, USA
15. Laboratoire de Biométrie et Biologie Evolutive, Centre National de la Recherche Scientifique, Université de Lyon, F-69622 Villeurbanne, France
16. Environmental Futures Research Institute, Griffith University Nathan 4111, Australia
17. Bioinformatics and Genomics Programme. Centre for Genomic Regulation (CRG), Dr. Aiguader 88, 08003 Barcelona, Spain
18. Universitat Pompeu Fabra, Barcelona, Spain
19. Institució Catalana de Recerca i Estudis Avançats, Barcelona, Spain
20. Joint Institute for Computational Sciences, The University of Tennessee, Oak Ridge National Laboratory, Oak Ridge, TN 37831 USA
21. Bioinformatics Research Centre, Aarhus University DK-8000 Aarhus C, Denmark
22. The Genome Institute, Washington University School of Medicine St Louis, MI USA
23. Department of Biochemistry, Molecular Biology, Entomology and Plant Pathology, Mississippi State University Mississippi State, MS 39762 USA
24. Institute for Genomics, Biocomputing and Biotechnology, Mississippi State University Mississippi State, MS 39762 USA
25. Department of Biological Sciences, Texas Tech University Lubbock TX, 79409 USA
26. Department of Ecology and Evolutionary Biology, University of California Santa Cruz, Santa Cruz, CA 95064 USA
27. Organisms and Environment Division, Cardiff School of Biosciences, Cardiff University Cardiff CF10 3AX, Wales, UK
28. Key Laboratory of Animal Ecology and Conservation Biology, Institute of Zoology, Chinese Academy of Sciences, Beijing 100101 China
29. International Wildlife Consultants, Ltd. Carmarthen SA33 5YL, Wales, UK
30. College of Medicine and Forensics, Xi'an Jiaotong University Xi'an, 710061, China
31. State Key Laboratory for Agrobiotechnology, China Agricultural University Beijing, 100094, China
32. Department of Ecology and Evolutionary Biology, Tulane University, New Orleans, LA 70118, USA.
33. Museum of Natural Science and Department of Biological Sciences, Louisiana State University Baton Rouge, LA 70803, USA
34. Center for Zoo and Wild Animal Health, Copenhagen Zoo Roskildevej 38, DK-2000 Frederiksberg, Denmark
35. Department of Behavioral Neuroscience Oregon Health & Science University Portland, OR 97239 USA
36. Brazilian Avian Genome Consortium (CNPq/FAPESPA-SISBIO Aves), Federal University of Para, Belem, Para, Brazil.
37. Institute of Biological Sciences, Federal University of Para, Belem, Para, Brazil
38. Institute of Medical Biochemistry Leopoldo de Meis, Federal University of Rio de Janeiro, Rio de Janeiro, RJ 21941-902, Brazil
39. Centre for Biological Sequence Analysis, Department of Systems Biology, Technical University of Denmark Kemitorvet 208, 2800 Kgs Lyngby, Denmark
40. Breeding Centre for Endangered Arabian Wildlife Sharjah, UAE
41. Dubai Falcon Hospital, Dubai, UAE
42. Canterbury Museum Rolleston Avenue, Christchurch 8050, New Zealand
43. Trace and Environmental DNA Laboratory Department of Environment and Agriculture, Curtin University, Perth, Western Australia, 6102, Australia
44. Department of Integrative Biology, University of California, Berkeley CA 94720 USA
45. Laboratory of Genomic Diversity, National Cancer Institute Frederick, MD 21702 USA
46. Institute of Molecular and Cellular Biology, SB RAS and Novosibirsk State University, Novosibirsk, Russia
47. Smithsonian Institution National Museum of Natural History, Washington D.C. 20013 USA
48. BGI-Shenzhen, Shenzhen 518083, China
49. Department of Biological Sciences, National University of Singapore, Republic of Singapore
50. Department of Vertebrate Zoology, National Museum of Natural History, Smithsonian Suitland, Maryland 20746 USA
51. Center for Macroecology, Evolution and Climate, Natural History Museum of Denmark, University of Copenhagen, Universitetsparken 15, DK-2100 Copenhagen O Denmark
52. Bell Museum of Natural History, University of Minnesota, Saint Paul, MN 55108 USA
53. Department of Life Sciences, Natural History Museum, Cromwell Road, London, SW7 5BD, U.K.
54. Department of Life Sciences, Imperial College London, Silwood Park campus, Ascot SL5 7PY, U.K.
55. Smithsonian Conservation Biology Institute, National Zoological Park, Front Royal, VA 22630 USA
56. Smithsonian Conservation Biology Institute, National Zoological Park, 20008, Washington, DC, USA
57. Theodosius Dobzhansky Center for Genome Bioinformatics St. Petersburg State University St. Petersburg, Russia 199004
58. Oceanographic Center, Nova Southeastern University, Ft Lauderdale, FL 33004 USA
59. Center for Biomolecular Science & Engineering, University of California Santa Cruz, Santa Cruz, CA 95064 USA
60. San Diego Zoo Institute for Conservation Research Escondido, CA 92027 USA
61. Department of Vertebrate Zoology, MRC-116, National Museum of Natural History, Smithsonian Institution, Washington, D.C., 20013,USA,
62. Department of Environmental Health Science, University of Georgia, Athens, Georgia, 30602, USA
63. Moore Laboratory of Zoology & Biology Department, Occidental College, Los Angeles, CA 90041 USA
64. Department of Genomics and Genetics, The Roslin Institute and Royal (Dick) School of Veterinary Studies, University of Edinburgh, Easter Bush Campus Midlothian EH25 9RG, UK
65. Swedish Species Information Centre, Swedish University of Agricultural Sciences Box 7007, SE-750 07 Uppsala, Sweden
66. Key Laboratory of Zoological Systematics and Evolution, Institute of Zoology, Chinese Academy of Sciences, Beijing 100101 China
67. Department of Organismic and Evolutionary Biology and Museum of Comparative Zoology, Harvard University, Cambridge, MA USA
68. Institute of Theoretical Informatics, Department of Informatics, Karlsruhe Institute of Technology D- 76131 Karlsruhe, Germany
69. Department of Biochemistry & Biophysics, University of California San Francisco, CA 94158 USA
70. Department of Ornithology, American Museum of Natural History New York, NY 10024 USA
71. Department of Biology and Genetics Institute, University of Florida, Gainesville, FL 32611 USA
72. Department of Biology, University of Copenhagen, Ole Maaløes Vej 5, 2200 Copenhagen, Denmark.
73. Princess Al Jawhara Center of Excellence in the Research of Hereditary Disorders, King Abdulaziz University, Jeddah 21589, Saudi Arabia.
74. Macau University of Science and Technology, Avenida Wai long, Taipa, Macau 999078, China
75. Department of Medicine, University of Hong Kong, Hong Kong
76. Centre for Social Evolution, Department of Biology, Universitetsparken 15, University of Copenhagen, DK-2100 Copenhagen, Denmark
